# Mind what you say—general and specific mechanisms for monitoring in speech production

**DOI:** 10.3389/fnhum.2014.00514

**Published:** 2014-07-21

**Authors:** Greig I. de Zubicaray, Robert J. Hartsuiker, Daniel J. Acheson

**Affiliations:** ^1^School of Psychology, University of QueenslandBrisbane, QLD, Australia; ^2^Department of Experimental Psychology, Ghent UniversityGhent, Belgium; ^3^Neurobiology of Language Department, Max Planck Institute for PsycholinguisticsNijmegen, Netherlands

**Keywords:** speech production and perception, lexical access, monitoring, attention, control

## Introduction

For most people, speech production is relatively effortless and error-free. Yet it has long been recognized that we need some type of control over what we are currently saying and what we plan to say. Precisely how we monitor our internal and external speech has been a topic of research interest for several decades. The predominant approach in psycholinguistics has assumed monitoring of both is accomplished via systems responsible for comprehending others' speech.

This special topic aimed to broaden the field, firstly by examining proposals that speech production might also engage more general systems, such as those involved in action monitoring. A second aim was to examine proposals for a production-specific, internal monitor. Both aims require that we also specify the nature of the representations subject to monitoring.

### Domain general mechanisms

Some of the first evidence to support a proposal of a domain general monitoring or attentional selection mechanism being engaged in speech production was provided by functional magnetic resonance imaging (fMRI) investigations of semantic interference effects in picture-naming paradigms. Those studies identified differential activity in the anterior cingulate cortex (ACC), noting similar activity had been observed in fMRI studies of picture-word (PWI), Stroop and manual interference paradigms (e.g., de Zubicaray et al., 2006). Piai et al. (2013) provide the first confirmatory evidence of a domain general, ACC involvement during performance of Stroop, semantic PWI and manual Simon tasks. Although PWI might be considered a generalization of Stroop-like interference effects (e.g., MacLeod, 1991), the Simon task elicits an interference effect in manual responding by manipulating the spatial location of target stimuli (e.g., a square or triangle) on congruent and incongruent trials. Thus, any overlap in ACC activity across the three tasks can be interpreted as reflecting a domain general mechanism.

Electrophysiological studies provide another source of evidence for a domain general fronto-central monitoring mechanism via both stimulus- and response-locked analyses (Riès et al., 2013; Trewartha and Phillips, 2013; Acheson and Hagoort, 2014). Using a phoneme substitution task, Trewartha and Phillips (2013) report an error-related negative potential (ERN) similar to that observed for manual actions. They conclude that speech errors are detected by a general conflict monitoring mechanism supported by the ACC. However, Acheson and Hagoort (2014) tested the domain generality assumption by directly comparing event-related potentials (ERPs) on tongue twister (TT) and Flanker tasks. The TT task required participants to repeatedly and rapidly read sequences of regular non-words. Despite observing similar ERNs, Acheson and Hagoort observed few correlations between behavioral or electrophysiological measures from the two tasks.

Together, the results from the above studies suggest that competition among lexical-level representations might engage a domain general mechanism in the ACC, while conflicting sub-lexical (i.e., phoneme) level representations might engage a different mechanism, either in another subdivision of the ACC or elsewhere in superior medial frontal cortex.

The working memory requirements of production paradigms might also be crucial for the engagement of domain general mechanisms. Riès et al. (2013) address this issue in patients with lesions of the lateral prefrontal cortex (DLPFC), with results indicating that picture naming might not require mechanisms mediated by this region, unless working memory load is increased, as per the verbal Simon task they employed. Crowther and Martin (2014) demonstrate that working memory is clearly involved in more complex production paradigms that manipulate both semantic context and item repetition, such as blocked cyclic naming, finding significant correlations with verbal memory span. In addition, they report the decreasing slope of naming latencies within cycles derives from a process of narrowing down available responses from the set of items within the block as the trials progress. This indicates participants monitor the item names they produce in order to perform the task. Further, they argue this reflects a strategic, task-specific process rather than a mechanism involved in word production in more naturalistic settings. Intriguingly, Riès et al.'s and Crowther and Martin's converging conclusions regarding the involvement of working memory in production are supported by a recent study by Wirth et al. (2011). The latter authors employed anodal transcranial direct current stimulation (atDCS), an electrical brain stimulation technique that induces more efficient neural processing at the stimulation site. They reported aTDCS applied over the left DLPFC reduced the semantic interference effect observed in the blocked cyclic naming paradigm.

### Common vs. distinct mechanisms for monitoring inner and overt speech

While the dominant approach to speech monitoring has invoked a general role for the comprehension system, more recent approaches have emphasized distinct mechanisms for monitoring of inner speech. Pickering and Garrod (2014), for example, adapt a well-established mechanism of forward models to provide an account of how a production-internal monitor might operate. Riès et al. (2013) provide electrophysiological evidence that could be considered consistent with both approaches: They observed a larger error-related response over the left temporal cortex that started before, and peaked around, vocal onset during picture naming. Conceivably, this result could reflect a process of computing discrepancies between planned and upcoming utterances *or* the perception of an erroneous pre-articulatory representation.

Gauvin et al. (2013) provide a direct comparison of perception and production modalities within-participant using eye- tracking in a visual-world paradigm. Their findings support the involvement of the speech comprehension system in monitoring the output of one's own speech in addition to that of others. However, they found no evidence of robust changes in eye-movements before or around production onset, leading them to conclude that the comprehension system is unlikely to be involved in the monitoring of inner speech. As the authors note, this result might reflect a lack of sensitivity of the paradigm to monitoring of inner speech. Alternatively, it might reflect something about the *nature* of inner speech. Lind et al. (in press) introduced a novel real-time speech exchange paradigm in which participants in a Stroop task occasionally received manipulated feedback of a single word they had spoken (i.e., they heard the name of the distractor word in their own voice, from an earlier recording). Surprisingly, the participants often considered the manipulated word as an error in their own production. Based on these data Lind et al. (2014) propose that inner speech might be relatively under-specified, and that auditory feedback of one's own voice has a more important role in monitoring as it provides a sense of agency in addition to verifying the intended meaning.

## Conclusion

The articles in this special topic have broadened the available evidence base by providing novel data from a range of behavioral, lesion, electrophysiological, and neuroimaging investigations. In addition, they have provided new theoretical perspectives on speech monitoring that future research will need to address. Results across the studies suggest that monitoring in language production is likely to involve not just a single mechanism, but multiple systems.

### Conflict of interest statement

The authors declare that the research was conducted in the absence of any commercial or financial relationships that could be construed as a potential conflict of interest.
